# Modelling in the light of uncertainty of key parameters: a call to exercise caution in field predictions of *Bt*-maize effects

**DOI:** 10.1098/rspb.2010.2085

**Published:** 2011-01-05

**Authors:** A. Lang, S. Brunzel, M. Dolek, M. Otto, B. Theißen

**Affiliations:** 1 Institute of Environmental Geosciences, University of Basel, Bernoullistrasse 30, 4056 Basel, Switzerland; 2 Faculty of Biology, Department of Animal Ecology, University of Marburg, Karl-v.-Frisch-Str., 8a, 35032 Marburg, Germany; 3 Büro Geyer und Dolek, Obere Dorfstr. 16, 82237 Wörthsee, Germany; 4 Federal Agency for Nature Conservation (BfN), Konstantinstrasse 110, 53179 Bonn, Germany; 5 gaiac – Research Institute for Ecosystem Analysis and Assessment at the RWTH Aachen University, Worringerweg 1, Aachen 52056, Germany

Perry *et al*. [1] developed a model to simulate the field exposure and adverse effects for three European non-target Lepidoptera species (*Inachis io* L., *Vanessa atalanta* L., and *Plutella xylostella* L.) to pollen of the *Bacillus thuringiensis* (*Bt*) maize MON810 containing lepidopteran-targeting Cry1Ab toxin. Perry *et al*. explicitly modelled the worst case scenario and came to hard quantitative predictions. However, the incomplete and uncertain input data cause a higher uncertainty than Perry *et al*. indicate, and we are specifically concerned with the possibility that the effects might be worse than they predict. Here, we specify this uncertainty by addressing some of the basic model assumptions and input data regarding the toxic effects of Cry1Ab to lepidopteran larvae. We do not address the hypotheses of the model regarding maize pollen dispersal and deposition, or population-dynamic effects.

A key problem of the modelling study of Perry *et al*. is that virtually no studies exist in the peer-reviewed literature reporting on the dose–response effects of MON810 pollen to European non-target Lepidoptera [2]. In consequence, the authors had to substitute data obtained from experiments with another transgenic event expressing Cry1Ab, the *Bt*176 maize [3,4]. In our view, the use of these studies is problematic for several reasons.

First, the authors set the toxicity of MON810 pollen to 31-fold less than *Bt*176 maize pollen, but did not provide data supporting the implied assumption of a linear relationship between Cry1Ab dose and adverse effects. The dose–effect relation may be nonlinear, possibly resulting in a disproportionally higher effect of low Cry1Ab concentrations [5,6].

Second, the assumption of a 31-fold difference in Cry1Ab concentration of pollen of MON810 and *Bt*176 is an average value, which Perry *et al*. seemingly derived from four papers (but three of them refer to the same source, the US Environmental Protection Agency). However, Cry1Ab content in pollen varies substantially even within the same event depending on the year, the site, the plant and possibly the cultivar [7]. For example, Cry1Ab concentration has been recorded to be as low as 389 ng g^−1^ in *Bt*176 pollen and as high as 97 ng g^−1^ in MON810 pollen, which is only a fourfold difference [8]. In consequence, the relevant information would be the Cry1Ab toxin concentration of the pollen used in the studies upon which the model is based; however, this was not quantified in these publications [3,4].

Third, Perry *et al*. claim that larvae of the red admiral, *Vanessa atalanta*, are equally susceptible to Cry1Ab as those of *Inachis io*. The evidence cited [9] did not test or report Cry1Ab susceptibility of *V. atalanta*, and indeed, no toxicological tests with Cry1Ab are published for this species so far. Susceptibility to *Bt* can vary greatly among lepidopteran species, e.g. 10-fold between close relatives within a genus, making a prediction of the sensitivity to *Bt* for any given species difficult [3,10], and extrapolating model data from one species to another may be inappropriate [11,12].

Fourth, the values for mortality incorporated into the model are likely to underestimate the real values owing to two experimental limitations in the studies used: Felke & Langenbruch [3] and Felke *et al*. [4] exposed the lepidopteran larvae to *Bt* maize pollen for 2 days and terminated the trials after 7 days. Exposure in the field is likely to last longer as maize fields shed pollen over a prolonged period, on average 8 days or even longer [13]. Also, the larvae will continue to suffer lethal and sublethal effects in later stages (older than 7 days) owing to long-term effects following a short acute dose of Cry1Ab [6]. Both will result in lower LC/EC_50_ values than applied in the model. Several other conditions of the above laboratory studies also do not reflect realistic field situations, which have the potential to underestimate a Cry1Ab effect [2], e.g. using cut leaf disks instead of whole host plants, providing ample food to the larvae, keeping larvae under favourable abiotic conditions and excluding multiple environmental stressors exacerbating a *Bt* effect, e.g. bacterial infections of lepidopteran larvae [14].

Acute toxicity may not be a reliable predictor of sublethal effects [15]. Owing to lack of data, Perry *et al*. assume sublethality rate to be four times the mortality rate. The justification of this assumed relationship remains to be clarified as the papers ‘broadly consistent’ with this assumption [4,6] do not allow deriving such a ratio of sublethal effects to mortality. For example, a *Bt* maize pollen density of 18.7 grains cm^−2^ caused a significant average reduction in body mass of caterpillars of the common swallowtail (*Papilio machaon*), while the same pollen density caused no increased mortality [6]. The notion of the authors that the reduction of larval body mass would be representative for all other types of sublethal parameters seems to lack conclusive support. Especially, effects on fecundity can be as important as effects on survival [16].

Any Cry1Ab effect will be moderated in the field by variation of exposure. However, all publications cited in Perry *et al*. in support of a possible reduction in exposure through behaviour of the larvae refer to *Danaus plexippus*, the American monarch butterfly, but none to the three modelled European species which show different (feeding) behaviours. Likewise, several other parameters were also set by personal (unreferenced) estimates. These include the proportion and density of host plants in and near maize fields, or the temporal overlap of larval occurrence and *Bt*-maize cultivation (see parameters in table 1 of Perry *et al*. [1]).

Perry *et al*. provided the first model study for *Bt*-maize effects on Lepidoptera. The assessment of such effects is complex, thus modelling approaches are welcomed. Model studies are especially helpful to better identify and understand complex interactions of key parameters and basic processes. However, risk assessment of genetically modified organisms is a sensitive area, and any quantitative conclusions should be drawn and published with greatest care, as these could have significant policy and regulatory implications. As specified above, the claim of Perry *et al*. to have simulated the worst-case scenario must be challenged. Moreover, their model study involves considerable uncertainty in values of key ingredients, and their chosen approach is not sufficiently cautious to reflect this uncertainty. A full uncertainty/sensitivity analysis would need to be performed before making detailed quantitative predictions, and certainly for predictions outside the original application.
